# Volunteer feedback and perceptions after participation in a phase I, first-in-human Ebola vaccine trial: An anonymous survey

**DOI:** 10.1371/journal.pone.0173148

**Published:** 2017-03-08

**Authors:** Julie-Anne Dayer, Claire-Anne Siegrist, Angela Huttner

**Affiliations:** 1 Division of Infectious Diseases, Geneva University Hospitals and Faculty of Medicine, Geneva, Switzerland; 2 Center for Vaccinology, Geneva University Hospitals and Faculty of Medicine, Geneva, Switzerland; 3 WHO Collaborating Centre for Vaccine Immunology, Faculty of Medicine, Geneva, Switzerland; 4 Infection Control Program, Geneva University Hospitals and Faculty of Medicine, Geneva, Switzerland; Universidad Peruana Cayetano Heredia, PERU

## Abstract

The continued participation of volunteers in clinical trials is crucial to advances in healthcare. Few data are available regarding the satisfaction and impressions of healthy volunteers after participation in phase I trials, many of which lead to unexpected adverse events. We report feedback from over 100 adult volunteers who took part in a first-in-human trial conducted in a high-income country testing an experimental Ebola vaccine causing significant reactogenicity, as well as unexpected arthritis in one fifth of participants. The anonymous, internet-based satisfaction survey was sent by email to all participants upon their completion of this one-year trial; it asked 24 questions concerning volunteers’ motivations, impressions of the trial experience, and overall satisfaction. Answers were summarized using descriptive statistics. Of the 115 trial participants, 103 (90%) filled out the survey. Fifty-five respondents (53%) were male. Thirty-five respondents (34%) were healthcare workers, many of whom would deploy to Ebola-affected countries. All respondents cited scientific advancement as their chief motivation for participation, while 100/103 (97%) and 61/103 (59%) reported additional “humanitarian reasons” and potential protection from Ebolavirus, respectively. Although investigators had documented adverse events in 97% of trial participants, only 74 of 103 respondents (72%) recalled experiencing an adverse event. All reported an overall positive experience, and 93/103 (90%) a willingness to participate in future trials. Given the high level of satisfaction, no significant associations could be detected between trial experiences and satisfaction, even among respondents reporting adverse events lasting weeks or months. Despite considerable reactogenicity and unexpected vaccine-related arthritis, all survey respondents reported overall satisfaction. While this trial’s context was unique, the positive feedback is likely due at least in part to the intense communication of trial information to participants, which included both general findings and personalized results.

## Introduction

The continued participation of volunteers in clinical studies, particularly phase I trials, is critical to advances in healthcare. Most information on participants’ motivating factors, satisfaction levels and overall impressions comes from patients enrolled in oncologic, surgical and HIV studies [1]; few data are available regarding healthy volunteers’ impressions after participation in phase I trials. Such information takes on greater importance in light of the lay media’s extensive coverage of phase I failures such as the recent “disasters” in Rennes, France [2], and London, UK [3].

Under the coordination of the World Health Organization, we recently completed a first-in-human, phase I/II randomized, double-blind, placebo-controlled trial to test the safety and immunogenicity of the recombinant vesicular stomatitis virus-vectored Zaire Ebola vaccine (rVSV-ZEBOV) in 115 healthy adult volunteers (clinicaltrials.gov identifier NCT02287480) [4, 5]. This live, replication-competent vaccine caused dose-dependent reactogenicity (flu-like symptoms) in 93% of vaccinees, as well as unexpected, dose-independent arthritis and/or dermatitis in 25%. The study, conducted entirely in Geneva, Switzerland, is described in detail elsewhere [4, 5]; briefly, it began in November 2014 at the height of the Ebola epidemic and included volunteers who were either “deployable” (planning to deploy to Ebola-affected countries) or “non-deployable” to undergo a single injection of either 10 million or 50 million plaque-forming units (pfu) of rVSV-ZEBOV or placebo. A study hold was called four weeks after study launch after the unexpected observation of arthritis in several participants; at this time 59 volunteers had received either 10 or 50 million pfu of rVSV-ZEBOV (n = 35 and n = 16, respectively), or placebo (n = 8). All participants were contacted by telephone with this news and were solicited regarding joint complaints. The study interruption received international media coverage given the trial’s high profile.

As the arthritis proved to be self-limited and was not seen in other parallel studies testing lower doses [4, 6], the study resumed in January 2015 to test a single, sharply reduced dose (300,000 pfu); ultimately 51 volunteers received this dose and five placebo, bringing the final sample size to 102 vaccinees and 13 placebo recipients, i.e. the planned 115 volunteers. While both reactogenicity and immune responses were significantly less intense at the lowest dose, vaccine-related arthritis proved dose-independent: ultimately 24 cases of arthritis were diagnosed (11/51 in the high-dose and 13/51 in the low-dose groups), some with associated vaccine-related dermatitis. With its resumption, the study’s follow-up period was extended from six to 12 months for all vaccinees. All participants received a remuneration of 90 CHF (92 USD) per visit; vaccinees, who attended 10 visits over 12 months thus received 900 CHF (6% of the country’s annual minimum wage), and placebo recipients, who attended nine visits over six months, received 810 CHF (5% of minimum wage).

Some unusual features of the vaccine trial were its high-profile media context, its population (roughly one third work in the healthcare sector), and the communication of individual results to each participant midway through and at the end of the trial. The study launched at the height of the Ebola epidemic: little active recruitment was necessary, and only two volunteers were lost to follow-up due to relocation abroad. The blind was lifted for participants with arthritis during their clinical work-ups, and by April 2015 for all volunteers. At the six- and 12-month visits, each participant received personalized information on his or her antibody responses to the vaccine.

Given the unexpectedly high proportion of subjects with vaccine-induced adverse events, which lasted between 1–2 days (early reactogenicity) and several months (arthritis; median 17 days, interquartile range [IQR] 7–71), the objective of the present survey was to assess the perceptions of our volunteers anonymously, including their level of satisfaction, and obtain their feedback on the conduct of the trial.

## Materials and methods

An anonymous, internet-based survey was constructed using www.surveymonkey.com (Surveymonkey Inc., Palo Alto, CA); an invitation to complete the survey was sent by email to all 115 participants of the trial one year after injection. The survey was available in English and French and consisted of 24 questions, both multiple-choice and open-ended, divided into four sections (S1 File). The first section covered general characteristics of respondents; the second, their impressions of the trial’s conduct; the third, their impressions regarding adverse events; and the fourth, their final overall perceptions. Level of satisfaction at the end of study (question no. 21) was this survey study’s primary outcome. Likert scales were used to grade answers, from 1 (strongly agree) to 7 (strongly disagree). No remuneration could be offered for survey participation.

Data were extracted from http://www.surveymonkey.com and analyzed in Stata, Release 14 (StataCorp LP, College Station, TX). Continuous data are presented as the mean (± standard deviation [SD]) unless otherwise specified. Categorical data are presented as counts and/or percentages. Comparisons between groups used an independent Student’s t-test for continuous data and a Chi^2^ or Fisher’s exact test, as appropriate, for categorical data. Univariable logistic regression models were planned to evaluate associations among baseline characteristics (treatment arm, adverse event status) and level of satisfaction. Associations with *P* values of ≤0.05 were considered statistically significant.

This survey and its implementation were approved by the local Ethics Committee (Canton of Geneva, approval no. 14–221) and that of the World Health Organization (no. RPC-696).

## Results and discussion

### Respondents’ baseline characteristics

**Fig 1:**
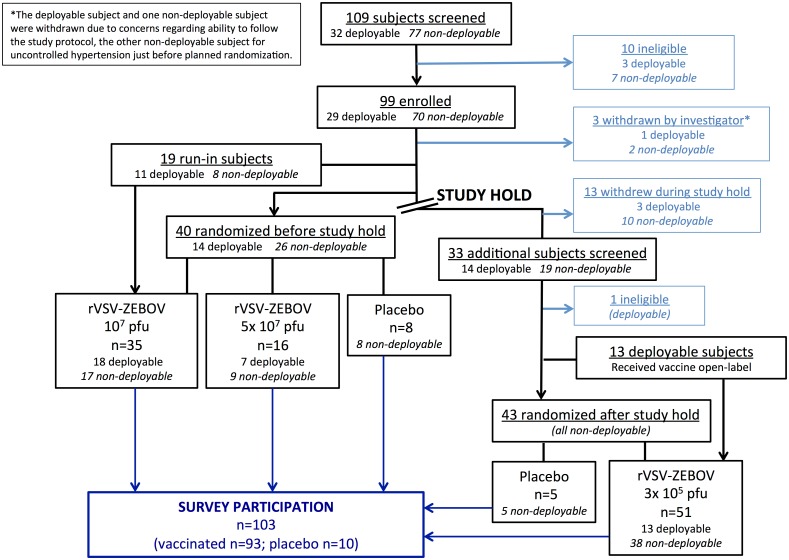
The trial flowchart with corresponding numbers of trial and survey participants. Because the survey was conducted anonymously, exact doses received by vaccinee respondents are unknown.

Of the 115 trial volunteers, 103 (90%) participated in the anonymous survey; 93/102 (91%) vaccinees and 10/13 (77%) placebo recipients responded (Fig 1). All respondents answered every survey question (see S1 File for the compilation of all answers). Respondents’ baseline characteristics are listed in Table 1; 55/103 (53%) were male, the most frequent age category was 46–55 years, and 82/103 (80%) were university-educated. Thirty-five respondents (34%) were healthcare workers; 24/103 (23%) worked for an international organization. One third reported employment at the University Hospitals of Geneva (HUG). Among the 93 vaccinees, 64 respondents reported receiving the “lowest dose,” when in fact only 51 participants received 300,000 pfu: this likely includes responses from vaccinees who had received 10 million pfu, since before the study hold, they were indeed receiving the “lowest dose.” All respondents were motivated to participate by a desire to further scientific progress. Fifteen of the 42 respondents reporting additional financial motivation were students: they were 7.9 times (95% confidence intervals [CI] 2.4–26.1) more likely than others to report financial interest.

**Table 1:** Baseline characteristics reported by respondents. Of 115 trial volunteers, 103 (90%) completed the survey.

**Variable**	**Number (%)**
**Sex**	
**Male**	55 (53)
**Female**	48 (47)
**Age**	
**18–25**	15 (15)
**26–35**	24 (23)
**36–45**	24 (23)
**46–55**	29 (28)
**56–65**	11 (11)
**Education level**	
**Obligatory school**	1 (1)
**Secondary school general**	6 (6)
**Secondary school professional**	3 (3)
**Higher professional**	11 (11)
**University**	82 (80)
**Profession**	
**Student**	19 (18)
**Healthcare worker**	35 (34)
**Hospital employee**	34 (33)
**International organization**	24 (23)
**Other worker**	20 (19)
**Trial treatment arm**	
**High-dose vaccine (10 or 50 million pfu)**	29 (28)
**Low-dose vaccine (300,000 pfu)**	64 (62)
**Placebo (normal saline)**	10 (10)
**Reason(s) for trial participation**	
**Contribution to scientific progress**	103 (100)
**Humanitarian concerns**	100 (97)
**Protection from Ebolavirus disease**	61 (59)
**Financial benefit**	42 (41)
**To support a loved one**	41 (40)
Other*	21 (20)
**Adverse events during the trial**	
**Reported experiencing pain and swelling in one or more joints**	37 (36)
**Reported developing vaccine-related skin lesions**	18 (17)
**Side effects diminished quality of life for a period of:**	
• **Days**	32 (31)
• **Weeks**	10 (10)
• **Months**	7 (7)
• **Did not have side effects**	54 (52)
**Variable**	**Number (%)**
**Sex**	
**Male**	55 (53)
**Female**	48 (47)
**Age**	
**18–25**	15 (15)
**26–35**	24 (23)
**36–45**	24 (23)
**46–55**	29 (28)
**56–65**	11 (11)
**Education level**	
**Obligatory school**	1 (1)
**Secondary school general**	6 (6)
**Secondary school professional**	3 (3)
**Higher professional**	11 (11)
**University**	82 (80)
**Profession**	
**Student**	19 (18)
**Healthcare worker**	35 (34)
**Hospital employee**	34 (33)
**International organization**	24 (23)
**Other worker**	20 (19)
**Trial treatment arm**	
**High-dose vaccine (10 or 50 million pfu)**	29 (28)
**Low-dose vaccine (300,000 pfu)**	64 (62)
**Placebo (normal saline)**	10 (10)
**Reason(s) for trial participation**	
**Contribution to scientific progress**	103 (100)
**Humanitarian concerns**	100 (97)
**Protection from Ebolavirus disease**	61 (59)
**Financial benefit**	42 (41)
**To support a loved one**	41 (40)
Other*	21 (20)
**Adverse events during the trial**	
**Reported experiencing pain and swelling in one or more joints**	37 (36)
**Reported developing vaccine-related skin lesions**	18 (17)
**Side effects diminished quality of life for a period of:**	
• **Days**	32 (31)
• **Weeks**	10 (10)
• **Months**	7 (7)
• **Did not have side effects**	54 (52)

*See S1 File.

### Trial conduct evaluation by respondents

Respondents’ views regarding trial conduct are described in Table 2. All survey participants reported being given enough time to read the informed consent form (ICF), available in both English and French, and to ask questions about the study before signing it; one respondent (1%) was unsatisfied by investigators’ answers. Five (5%) volunteers had difficulty understanding the ICF. All reported being treated with respect and being clearly informed throughout the trial. Five (5%) respondents deemed the remuneration inadequate; four of these also reported joint swelling (*P* = 0.055).

**Table 2:** Survey respondents’ perceptions of the trial’s conduct. Detailed responses including free-text comments can be found online (S1 File).

Survey question	Responses (%)						
	Strongly agree	Agree	Somewhat agree	Neutral	Somewhat disagree	Disagree	Strongly disagree
**I had enough time to read the informed consent brochure and to ask questions before signing the consent form.**	79 (77)	23 (22)	1 (1)	0 (0)	0 (0)	0 (0)	0 (0)
**The informed consent form was difficult to understand and increased confusion.**	3 (3)	1 (1)	1 (1)	9 (9)	3 (3)	38 (37)	48 (47)
Study personnel fully answered my questions regarding the study and the informed consent form (or I did not have any questions regarding the study or informed consent form*).	79 (77)	17 (17)	0 (0)	0 (0)	0 (0)	1 (1)	0 (0)
**I was treated with respect at the study visits.**	95 (92)	7 (7)	1 (1)	0 (0)	0 (0)	0 (0)	0 (0)
**Throughout the study, I was clearly informed of its future course.**	77 (75)	24 (23)	2 (2)	0 (0)	0 (0)	0 (0)	0 (0)
**When I heard that there were non-severe, unexpected joint and skin side effects, this initially worried me and affected my daily well-being.**	0 (0)	7 (7)	7 (7)	14 (14)	2 (2)	36 (35)	37 (36)
If I had known about the side effects I would experience, I would not have participated in this study (or I did not experience any side effects**).	1 (1)	0 (0)	2 (2)	5 (5)	3 (3)	15 (15)	40 (39)
The side effects experienced are acceptable for a vaccine against Ebola virus disease (or I did not experience any side effects***).	38 (37)	24 (23)	8 (8)	3 (3)	0 (0)	0 (0)	0 (0)
**I had confidence in the study team and felt comfortable at study visits and (if applicable) during further work-ups that were not initially planned.**	78 (76)	22 (21)	2 (2)	1 (1)	0 (0)	0 (0)	0 (0)
**The remuneration for participation in this study is acceptable given the time I provided and the type and number of procedures I underwent.**	54 (52)	20 (19)	6 (6)	18 (17)	3 (3)	2 (2)	0 (0)
**Overall, my participation in this study has been a positive experience.**	68 (66)	31 (30)	4 (4)	0 (0)	0 (0)	0 (0)	0 (0)
**I would be willing to return in one year for a blood draw in order to evaluate the durability of the antibody response induced by the vaccine (or I received placebo**§**).**	75 (73)	19 (18)	0 (0)	0 (0)	0 (0)	0 (0)	1 (1)
**After this experience, I would be willing to participate in a future clinical trial.**	59 (57)	28 (27)	6 (6)	7 (7)	1 (1)	1 (1)	1 (1)

*Six respondents reported having no questions about the study or the informed consent form.

**In answers to this question, 37 respondents reported experiencing no side effects.

***In answers to this question, 30 respondents reported experiencing no side effects.

^§^Eight respondents received placebo.

#### Adverse events

Thirty of the 103 respondents (29%) reported experiencing no adverse event, a finding that contradicts trial results, as 97% of all trial participants experienced at least one event [5]. Thirty-seven (36%) and 18 (18%) respondents reported joint swelling and skin-related adverse events, respectively.

Only 14/103 (14%) respondents reported having been “concerned” by the unexpected emergence of adverse events; 12 of these had had joint swelling (*P*<0.001). At the same time, 49 (48%) respondents reported that study-related side effects had diminished their quality of life for days (n = 32), weeks (n = 10), or even months (n = 7).

#### Final overall participants’ perceptions and perspectives

All 103 respondents considered their trial participation to be an “overall positive” experience, and 93 (90%) reported a willingness to participate in future clinical trials. Among those who were neutral (n = 7) or unwilling (n = 3), six had joint- and/or skin-related adverse events. Of the 47 free-text comments left by respondents, 44 (94%) were positive, expressing compliments (n = 30) or gratitude (n = 27) for the study team’s work, and/or pride at having participated (n = 10). There were two negative comments (remuneration not disbursed on time, development of an anemia after frequent blood draws) and one neutral (recommendation for all trials to include an invitation in the informed consent for trial volunteers to view the final clinical study report). All respondent answers and free-text comments are available in S1 File. No differences in satisfaction were found between respondents reporting adverse events lasting months and those reporting events lasting only a few days.

Respondents were not asked to reveal their deployability status; stratification based on this criterion was impossible. Those working for an international organization (not all of whom were deployable) were thus examined as a subgroup: they were motivated significantly more by the hope of protection against Ebolavirus (19/24 [79%] vs. 42/79 [53%], *P* = .032) and less by financial compensation (3/21 [13%] vs. 39/79 [49%], *P* = .002). While there were no differences in reported overall satisfaction, only 75% of these respondents vs. 95% of local workers reported a willingness to participate in future trials (*P* = .010, question #23). We further compared the responses of HUG employees to others’: they did not differ in their reasons for trial participation, satisfaction levels, or willingness to participate in future trials. The only respondent reporting a non-positive (“neutral”) attitude regarding confidence levels in the study team and feeling comfortable at study visits (question #19) belonged to this group.

Given the high level of satisfaction among all respondents, univariable logistic regression models for determining associations between trial experiences and satisfaction could not be performed as planned.

## Discussion

The survey saw an unusually successful response rate of 90% and, despite unanticipated and relatively frequent adverse events often requiring extensive diagnostic work-ups, respondents’ overall satisfaction levels were robust. To our surprise, 90% of respondents reported a willingness to participate in further clinical trials.

This high satisfaction is likely due to several factors. First, many respondents were working in healthcare and/or for aid organizations at baseline and were highly motivated by a desire to further scientific progress. Second, there was a discrepancy between respondents’ reporting of adverse events and earlier realities: one year after injection, a significant proportion of volunteers did not even recall their adverse events. Thus the timing of the survey may have created a recall bias, which could, in turn, have boosted satisfaction levels. Third, the trial’s context and mission were not typical. The study launched at the height of the Ebola epidemic and received local and national attention, while in healthcare circles, the rVSV-ZEBOV vaccine was known to be a promising candidate. The trial was investigator-initiated and its study product perceived as potentially destined for underprivileged populations. Fourth, the reported high protective efficacy of the vaccine candidate in a ring-vaccination trial in Guinea [7], announced between the 6- and 12-month visits, and its selection for further testing in phase III trials in Africa may have introduced a “halo effect” [8], prompting respondents to subconsciously minimize earlier dissatisfaction.

Finally, the study team made communication with volunteers a high priority. The latter received personalized notification at the time of identification of the first cases of arthritis, and study staff were available at all times by email and phone or on site for questions and concerns. Indeed, all 103 respondents reported that, throughout the trial, they were “clearly informed” of its future course.

At the same time, the unblinding allowed all trial vaccinees to receive personalized information on their own immune responses to vaccination at both the six and 12 months visits, while the 13 placebo recipients were informed of their treatment allocation. The 2015 Report on Participation Experiences from the Center for Information and Study on Clinical Research Participation (CISCRP) found that 91% of trial volunteers find receiving even a general summary of study results important after a study ends, yet 51% never received any information after their participation ended [9]. We had expected substantially lower satisfaction rates given the widespread reactogenicity and the unexpected vaccine-related arthritis and/or dermatitis in a quarter of the trial population leading to the trial’s abrupt interruption; we surmise that the close communication and personalized feedback were critically mitigating factors.

This survey study has limitations. As with all surveys, a selection bias cannot be avoided and those willing to answer 24 questions were likely positively disposed to the study and its investigators. Participation was unusually high, however, with only 12/115 (10%) volunteers not responding. Many respondents were healthcare workers and as such might be, at baseline, more positively disposed toward “advancing science.” A third of respondents were employees of HUG, which raises the possibility of some perceived, undue influence on both willingness to participate and the nature of responses. While such an influence can never be ruled out, this group’s answers did not differ from those of non-employees. No trial volunteer was employed by study investigators before or during the trial; HUG employs roughly 11,000 people in its network of eight geographically separate hospitals and 40 clinics throughout the canton. The passage of a year between trial inclusion and survey conduction could have allowed misclassification of adverse events: a few respondents appear to have reported early post-vaccination joint pains and injection-site erythema as arthritis and disseminated skin involvement, respectively. While the trial’s unusual context, including its relatively high-income setting, may decrease the generalizability of the survey’s findings, the importance of regular and detailed communication between investigators and trial volunteers is applicable across all fields and settings.

In conclusion, all 103 survey respondents reported satisfaction with their participation in this phase I Ebola vaccine trial despite the occurrence of frequent and occasionally worrying adverse events. The high satisfaction is likely due to the close communication between investigators and volunteers, which included the transmission of personalized study results to each participant.

## Supporting information

S1 File This file contains the exact questions read by respondents as well as their answers, in tabulated and original free-text form. (PDF)
