# Supplementary material for: Volunteer feedback and perceptions after participation in a phase I, first-in-human Ebola vaccine trial: An anonymous survey
Source: PLoS One. 2017 Mar 8;12(3):e0173148. doi: 10.1371/journal.pone.0173148 (PMC5342214; doi:10.1371/journal.pone.0173148)
Supplement: S1 File — (PDF) [file pone.0173148.s001.pdf]

SUPPLEMENTARY MATERIAL

Volunteer feedback and perceptions after participation in a phase I, first-in-human Ebola vaccine trial: an anonymous survey

Julie-Anne Dayer, M.D.[1], Claire-Anne Siegrist[2,3], Angela Huttner[1,2,4]*

1 Division of Infectious Diseases, Geneva University Hospitals and Faculty of Medicine, Geneva, Switzerland

2 Center for Vaccinology, Geneva University Hospitals and Faculty of Medicine, Geneva, Switzerland

3 WHO Collaborating Centre for Vaccine Immunology, Faculty of Medicine, Geneva, Switzerland

4 Infection Control Program, Geneva University Hospitals and Faculty of Medicine, Geneva, Switzerland

*Corresponding author:

Angela Huttner

Division of Infectious Diseases

Geneva University Hospitals

Rue Gabrielle-Perret-Gentil 4

Geneva, Switzerland

angela.huttner@hcuge.ch

Tel. +41 22 372 2959

Below are the exact questions read by respondents as well as their answers, in tabulated and original free-text form.

1. I am:

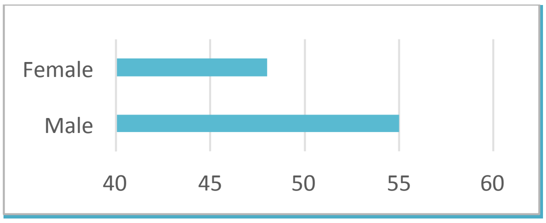

2. At the time of study enrollment, I was (years old):

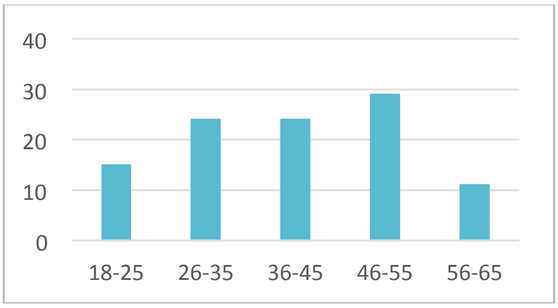

3. I received:

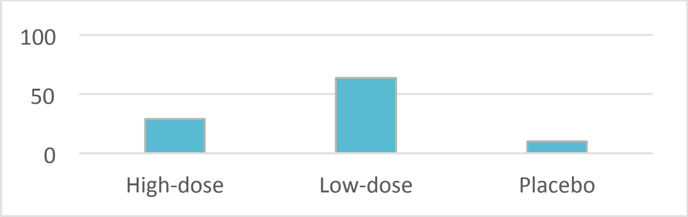

4. I am (multiple responses possible):

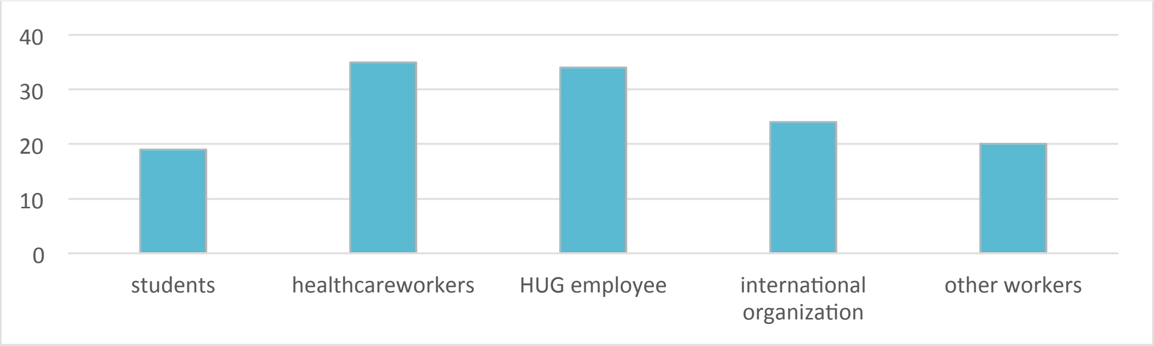

5. I completed:

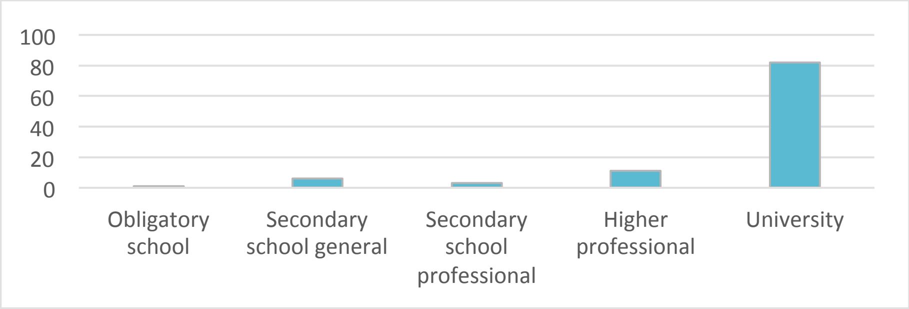

6. My reason(s) for participation in the study are the following (in order of importance, 1 being the most important):

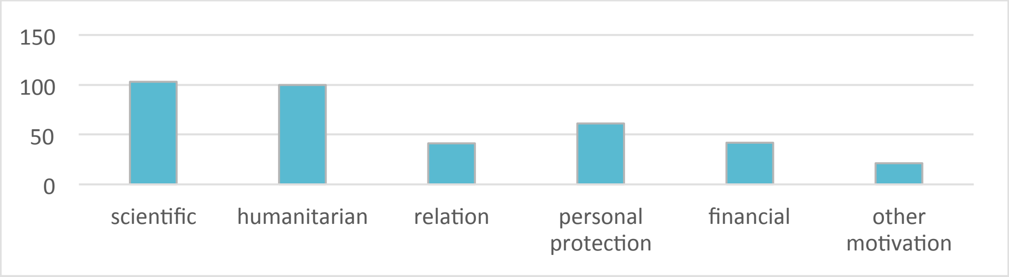

Free-text responses (motivation):

1) montrer l'exemple à mes enfants, aux étudiants en médecine etc
2) Obligation morale de participer à la recherche clinique scientique en général (pas seulement Ebola)
3) Dans le cadre de mon activité professionnelle je m'intéresse aux aspects éthiques des essais cliniques, notamment ceux menés dans des contextes / avec des personnes vulnérables (pays en développement)
4) Reportage
5) pour démontrer entourage importance de s'investir dans la Recherche en général!
6) Afin de voir de l'intérieur comment est-ce qu'un vaccin est testé.
7) Intérêt pour ces types de virus
8) Parce que je savais que ce serait déterminant pour lutter contre a maladie, notamment en permettant au personnel soignant effrayé de se protéger et de mieux intervenir.
9) J'ai habité au Libéria pendant 18 mois, je leur devais bien ça :)
10) Dans mon domaine de compétences, l'expérimentation animale est l'un des outils que j'utilise. Par ma participation, j'ai la possibilité de me retrouver pour une fois dans la situation du sujet de l'expérimentation.
11) Interested in biology/medicine and learning more about the process of a drug trial

7. My decision to participate in the study could have been influenced by (choose the correct option(s) and place in order of importance):

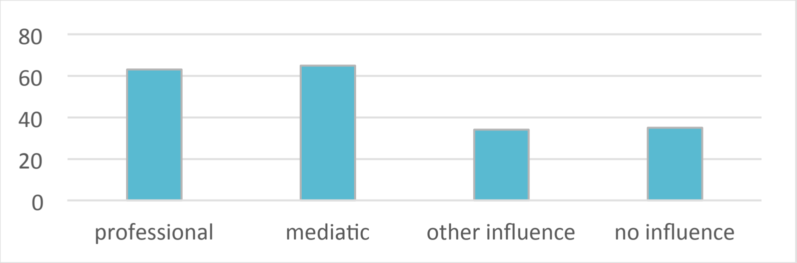

Free-text responses (influences):

1) Aider des collègues
2) Mes expériences antérieures dans l'aide médicale humanitaire
3) La très forte mobilisation de mes autres collègues (MSF) pour cette épidémie, et le fait que je ne pouvais pas, moi, m'impliquer dans la riposte via mon travail actuel.
4) Une amie dans le secteur humanitaire en Afrique m'a décrit la situation sur place.
5) Environnement MSF et académique
6) Par solidarité humaine et ma motivation.
7) J'ai participé d'abord à titre personnel (solidarité), mais aussi de par mon activité professionnelle (cf. 7)
8) L'atteinte de proches par Ebola
9) I have been working in previous Ebola outbreaks with case fatality rates between 70-90%
10) Par l'ampleur et la rapidité de l'évolution de l'épidémie et de l'urgence de faire valider un vaccin un vaccin.
11) évidence commandée par l'amour du prochain
12) la nécessité de répondre présent face à l'adversité rencontrée par d'autres. Sentiment que civisme et acte citoyen
13) Le fait de faire de la recherche et de toujours trouver des volontaires pour mes projets. Une opportunité de rendre/contribuer quelque chose.
14) Dans mon entourage, beaucoup de personne travaille dans le domaine de la médecine ou étudie à médecine. C'est grâce à ces personnes que j'ai entendu parler de cette étude.
15) Ma copine
16) influence: être déployable a influencé beaucoup. J'aurais bien voulu participer quand-même, mais à Lausanne les postes étaiet pleins très vites. A Genève j'avais de la chance avec la mission en février en Sierra Leone.
17) entourage
18) ma famille
19) Compagne dans le domaine de la santé
20) Je milite dans une association de solidarité entre la Suisse et la Guinée. je me sens particulièrement concerné car nos partenaires associatifs guinéens sont directement menacés.
21) Présentation de l'étude lors d'une réunion sur Ebola à l'OMS. Sinon je ne suis pas sure que j'aurais su que cette étude cherchait des participants.
22) Un intérêt pour le sujet en général
23) The main reason why I did the vaccine trial was that I was going to Sierra Leone to set up a charity and my family and friends were worried about me going there while the ebola epidemic was happening.I participated in the trial to put their minds at ease.
24) a sense of urgency that "we" = the Western world could at least contribute something substantially by investing time, money and energy to perform the study
25) Friends and family concerned about the risks
26) Intrinsic motivation - which I suppose you can ascribe to my background/upbringing
The fact that we could contribute personally to assist in eradicating the disease.

8. I had enough time to read the informed consent brochure and to ask questions before signing the consent form.

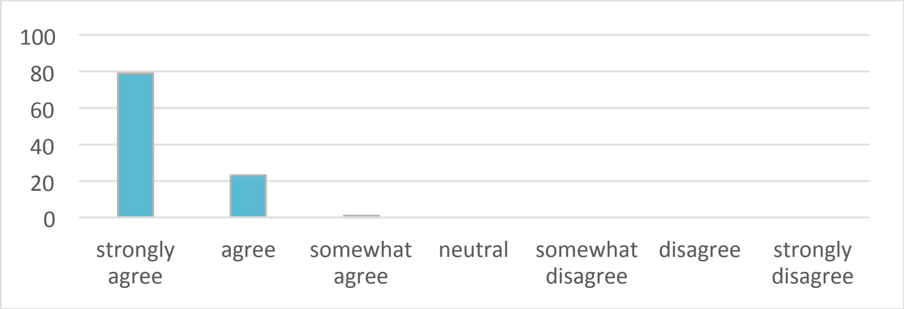

9. The informed consent form was difficult to understand and increased confusion.

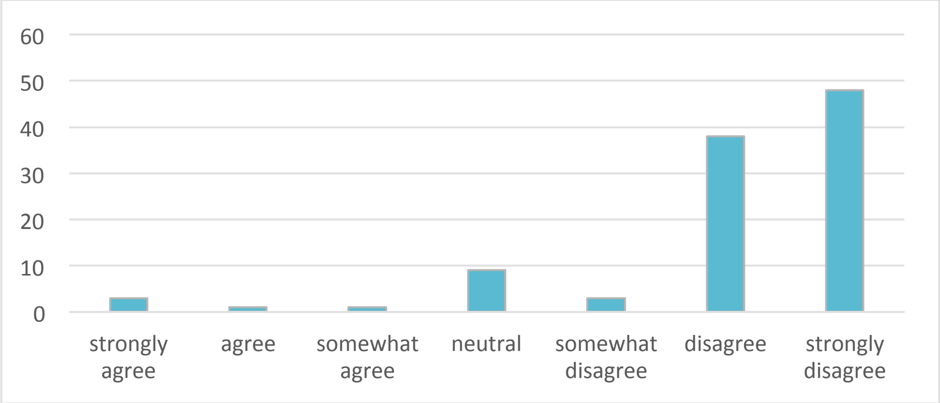

10. Study personnel fully answered my questions regarding the study and the informed consent form (or I did not have any questions regarding the study or informed consent form).

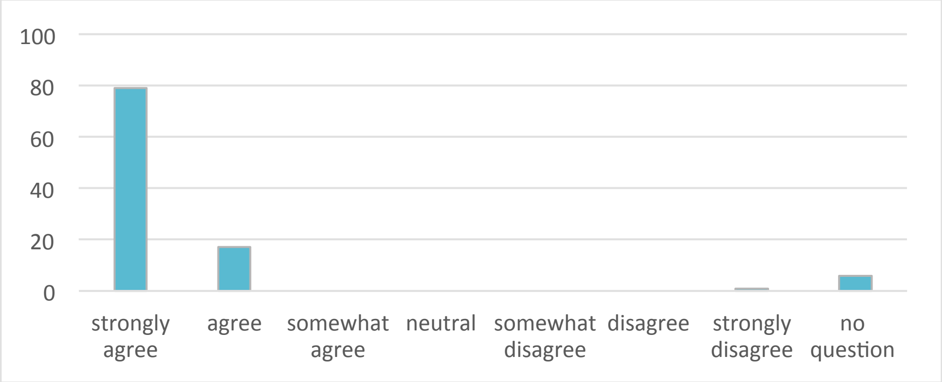

11. I was treated with respect at the study visits.

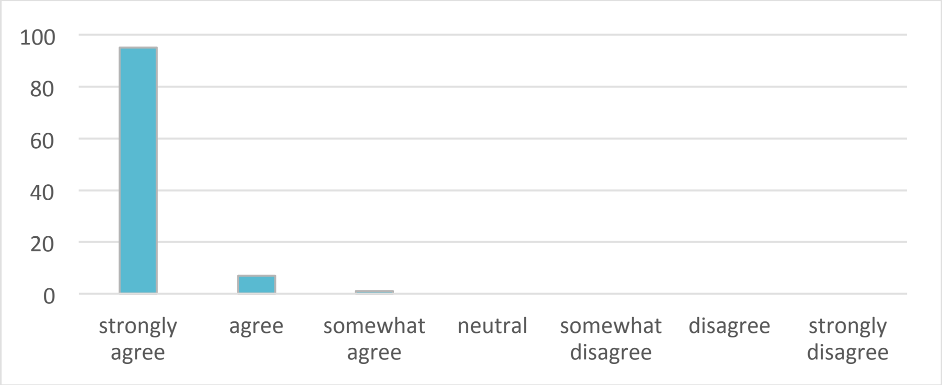

12. Throughout the study, I was clearly informed of its future course.

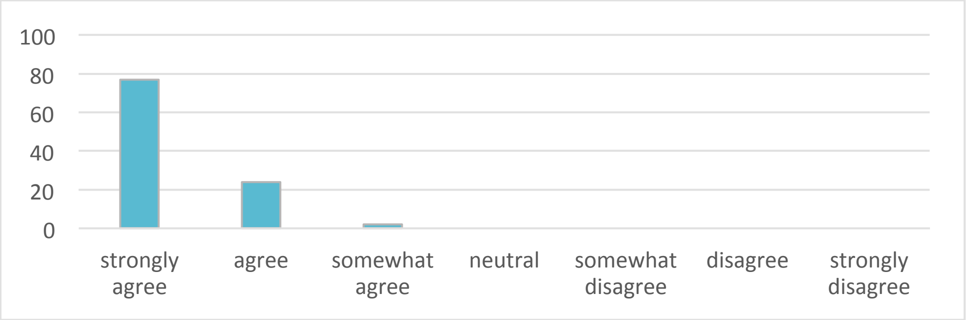

13. When I heard that there were non-severe, unexpected joint and skin side effects, this initially worried me and affected my daily well-being.

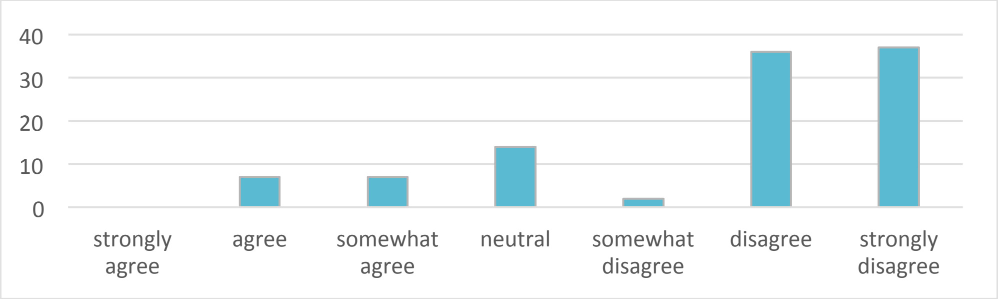

14. During the study, I had pain and swelling in one or more joints.

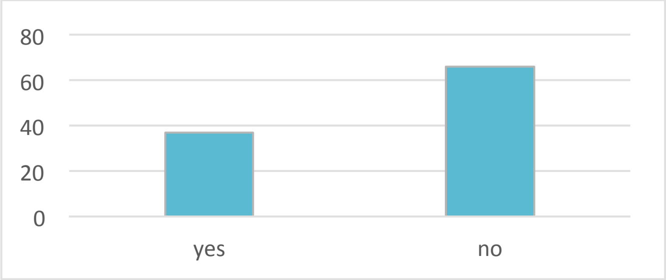

15. During the study, I developed skin lesions (presumed to be related to the vaccine).

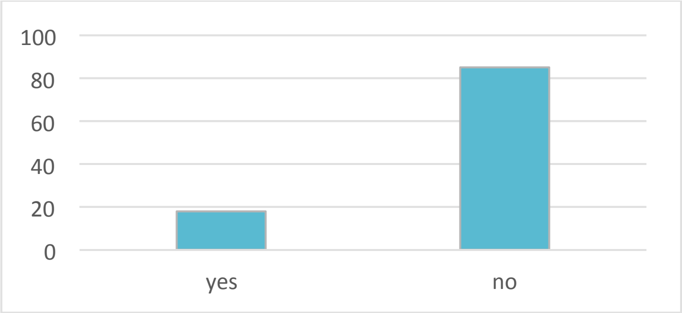

16. The side effects I experienced diminished my quality of life over a period of:*

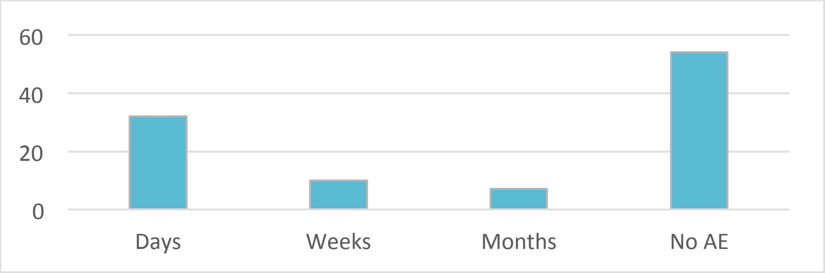

17. The side effects experienced are acceptable for a vaccine against Ebola virus disease or I did not experience any side effects.*

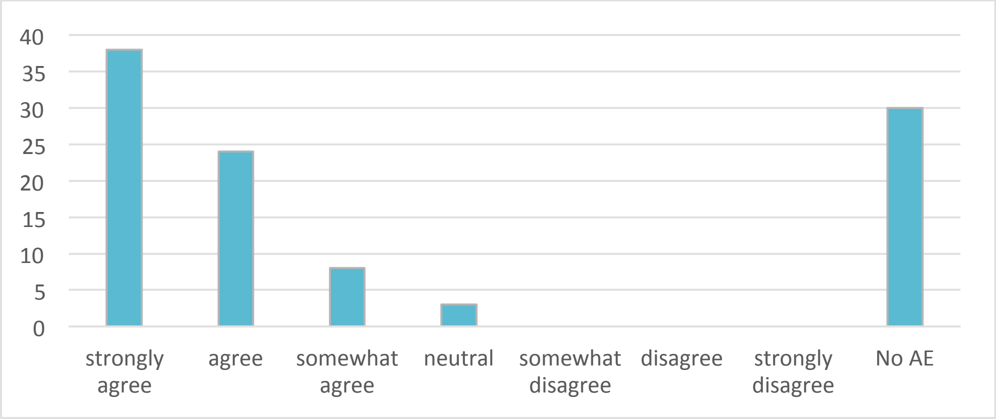

18. If I had known about the side effects I would experience, I would not have participated in this study or I did not experience any side effects.*

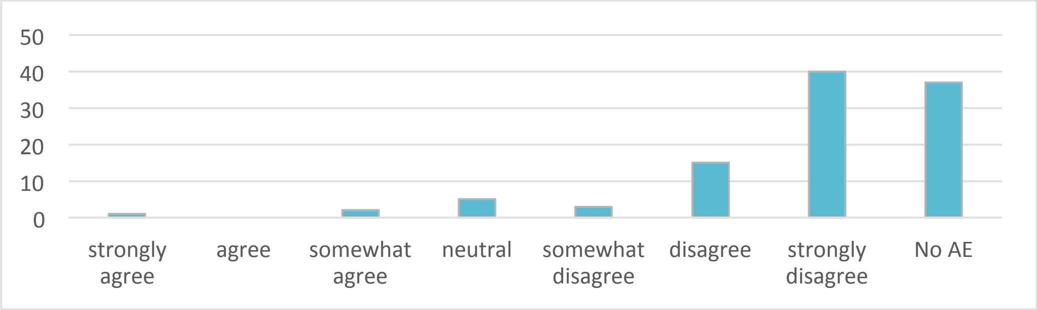

**Authors' note: We note a discrepancy in the number of respondents reporting "no adverse events" in answer to these three questions. For question 16, we speculate that some respondents may have felt obliged to choose the response "No AE" because they were not provided an option for AE that either did not diminish their quality of life at all or did so for a period of less than one day (in many cases, vaccine reactogenicity lasted for less than 24 hours). For the smaller discrepancy seen in the responses to questions 17 and 18, we can surmise that "survey fatigue" may have led a few respondents to report "No AE," but this remains speculation.*

19. I had confidence in the study team and felt comfortable at study visits and (if applicable) during further work-ups that were not initially planned.

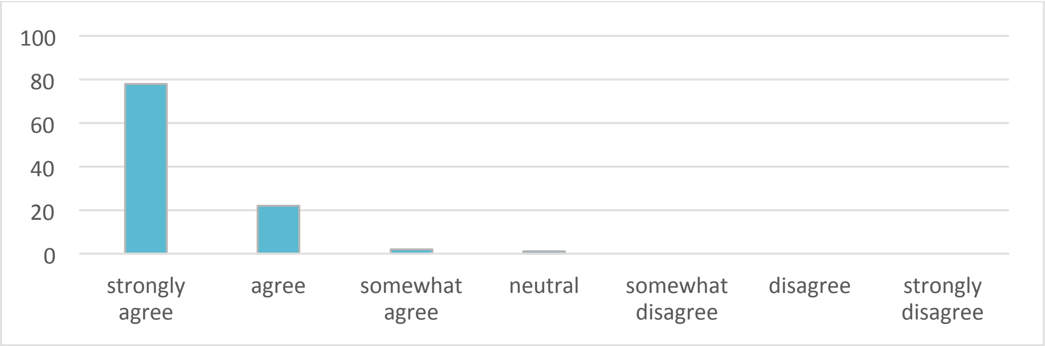

20. The remuneration for participation in this study is acceptable given the time I provided and the type and number of procedures I underwent.

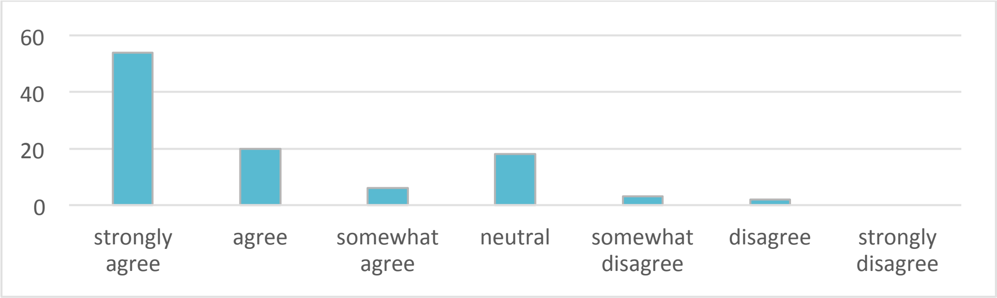

21. Overall, my participation in this study has been a positive experience.

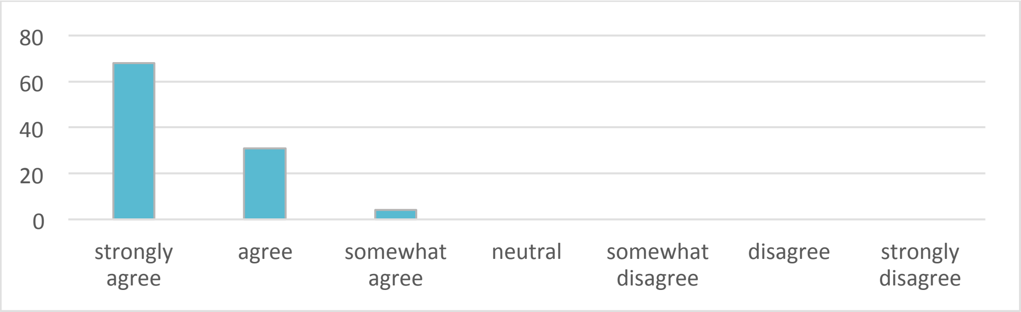

22. I would be willing to return in one year for a blood draw in order to evaluate the durability of the antibody response induced by the vaccine (or I received placebo).

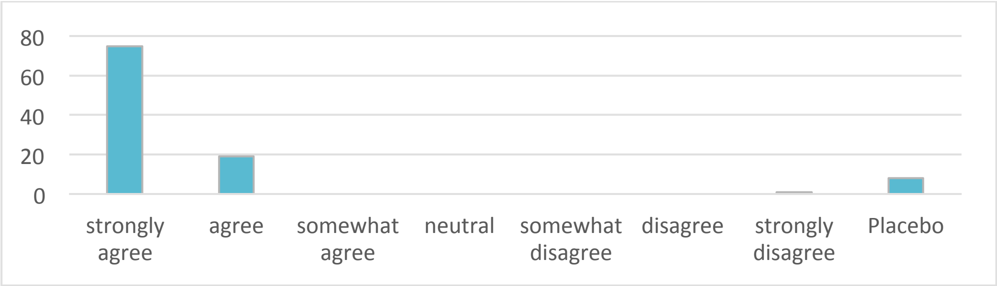

23. After this experience, I would be willing to participate in a future clinical trial.

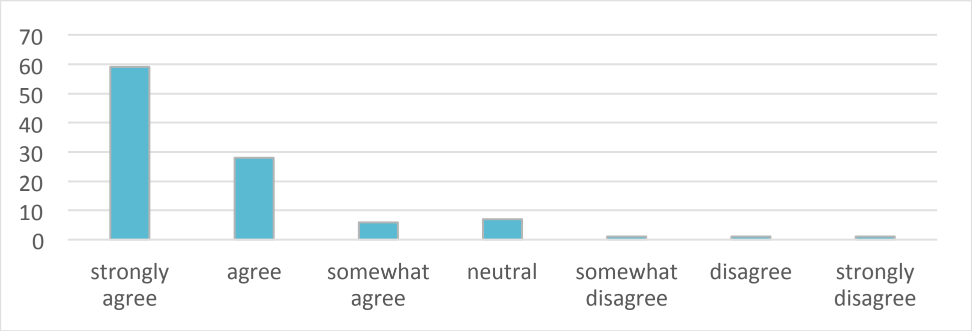

24. What I consider important for you to know:

1) Merci pour l'engagement sans faille de tout le team durant l'ensemble de l'étude.
2) Merci pour votre boulot, les cafés, et les sourires LK
3) Cela a été une expérience enrichissante et je me sens fière d'avoir pu aidé la science
4) Merci pour votre engagement et votre gentillesse.
5) Je suis très contente d'avoir pu participer à cette étude, d'autant plus qu'elle semble être utile dans la lutte contre l'épidémie d'Ebola. Merci à tous les collaborateurs pour le gentillesse et leur professionnalisme!
6) Tout s'est parfaitement déroulé. Le personnel est exemplaire.
7) C'est banal mais .... MERCI !
8) C'est pour moi , une belle expérience de vie et de solidarité humanitaire. Je trouve qu'il y a un bon encadrement de votre part ; cela rassure. Merci
9) -MERCI- PS: peut-être que mes fibres musculaires n'ont pas toute aimé cet intrus de VSV...et si nous faisions une biopsie, comme ça juste pour voir?
10) Chaque participant/e a certes obtenu une information générale (dose de vaccin reçue, résultats (très) généraux de l'étude) mais il devrait avoir la possibilité d'accéder au rapport complet de l'étude anonymisé (clinical study report) s'il le souhaite. Cela devrait être prévu dans le formulaire de consentement initial.
11) je vous félicite et me félicite pour cet investissement dans la participation à l'élaboration de ce projet humain et vous souhaite une bonne continuation
12) Well done, Congratulations!!
13) Merci pour cette prise en charge, et pour le travail accompli ainsi que pour l'espoir que vous avez apporté aux populations concernées.
14) Aide à au avancé de la médecine, je suis un homme de la planète terre.J AI BESOIN D AIDER MON PROCHAIN
15) bravo ! et merci !
16) J'ai développé une anémie suite aux prises de sang répétée dans le cadre de l'étude et je trouve navrant que l'équipe médicale n'a pas voulu me soigner pour ce problème surtout que je suis une employée des HUG. J'ai dû consulter mon médecin traitant pour le traitement.
17) l'accueil des professionnels est excellent et leur engagement est à souligner! Bonne suite au (x) projet(s)
18) Merci de faire avancer les recherches.
19) Vous avez une équipe formidable. Bravo pour ce magnifique projet qui nous rend fier de vivre à Genève.
20) Merci à vous et félicitations pour les résultats obtenus et votre investissement très important pour cette étude montée au pied levé.
21) Bravo pour le déroulement de cette étude et les résultats !
22) Merci à toute l'équipe médicale! Vous aviez toujours un immense sourire lors des rendez-vous. Ça a toujours été un plaisir de venir vous voir!
23) Mes meilleurs remerciements pour votre accueil, votre gentillesse, disponibilité et professionnalisme. À bientôt...
24) la réussite de l'étude m'a semblé reposer sur la compétences de l'équipe

25) Merci pour vos explications, toujours très pédagogiques, ainsi que pour votre travail.
26) Bravo
27) C'est surtout parce que j'ai eu connaissance de cette étude que je me suis portée volontaire. Les autres essais cliniques ne sont à mon avis pas moins importants et mériteraient tout autant de communication!
28) Jolie doctoresse... ;)
29) never give up
30) J'ai été traitée de manière respectueuse mais ai trouvé extrêmement maladroit la protection imposée par Swissmedic lors de l'injection (lunettes, masque, etc.) Cela donne l'impression qu'on nous injecte quelque chose de toxique, contre lequel l'infirmier doit se protéger alors que nous y serons exposés. En plus je ne crois pas que cela soit extrêment utile pour protéger ... les vaches ;-)!
31) Rassurer par l'équipe de tout ce qui aurait pu arriver si j'avais eu le vaccin, Total confiance de la prise en charge possible. Un grand merci et toutes mes féclictations pour le travail effectué dans l'urgence et l'organisation qui a été au top.
32) Merci a toute l'équipe qui a été très professionnel.
33) L'equipe qui nous a reçu a été agréable et professionnelle
34) Merci
35) Vous avez tous été une équipe géniale, et vous avez les meilleurs faiseurs de prises de sang au monde :)
36) Le rappel des prises de sang quelques jours avant le rendez-vous ! L'accueil des infirmières était super !
37) Merci et bravo pour voztre travail!
38) Tout le travail accompli par vos services est une contribution importante aux valeurs et principes d'humanité que je souhaite voir se développer. C'est une réponse de solidarité avec celles et ceux qui souffrent de multiples maladies non éradiquées dans le Sud. Si de telle recherches pouvaient se concrétiser pour lutter contre le paludisme notamment,ce serait merveilleux !
39) Je souhaite que le candidat vaccin testé démontre son efficacité sur le terrain des épidémies.
40) Un énorme merci à l'équipe médicale pour l'accueil, les explications, leur disponibilité et gentillesse. Vous avez rendu ces visites très agréables. Les explications ont permis de se sentir engagé et concerné, et d'avancer ensemble dans cette aventure. Un tout grand merci à vous!
41) Thank you for the experience!
42) I just want tyo say thank you to all the doctors and nurses involved in the study. You were all so nice to me. I think you all do fantastic work!
43) My only constraint was the time taken -- but worth it, in retrospect. Delighted by vaccine's success.
44) Appreciate the very open communication you had with me on my own situation as well as that of the trial throughout the entire period.
45) A big THANK YOU to the staff of the HUG for your professionalism, ethical conduct, and commitment!
46) I got only an initial payment and when asked about the second one (thinking that I might have missed it) I was told I was to be paid in June and that has not happened (to my knowledge). I did not participate at all in the study because of money and would have done it without any payment the same, BUT if you offer compensation I would expect you to fulfill what you offered unless you can not pay for whatever reasons, but explain that.
47) I am very happy to have participated.
